# Association of plasma lipid metabolism profiles with overall survival for patients with gastric cancer undergoing gastrectomy based on ^1^H-NMR spectroscopy

**DOI:** 10.1186/s12986-023-00728-1

**Published:** 2023-02-07

**Authors:** Yaopeng Qiu, Zhou Xu, Qingfeng Xie, Renyi Zhang, Luyao Wang, Liying Zhao, Hao Liu

**Affiliations:** 1 Department of General Surgery, Nanfang Hospital, Southern Medical University, 1838 North Guangzhou Ave, Guangzhou, 510515 China; 2 Guangdong IFV Biomedical Technology Co., Ltd, Foshan, China

**Keywords:** ^1^H-NMR, Gastric cancer, Plasma lipid metabolism profiles, Overall survival

## Abstract

**Background:**

Lipid metabolism dysregulation is a prominent metabolic alteration in various cancers. The study aimed to explore the association of plasma lipid metabolism profiles with overall survival (OS) for gastric cancer (GC) patients who received gastrectomy.

**Methods:**

GC patients who were treated with gastrectomy and measured with plasma lipid metabolism profiles using proton nuclear magnetic resonance (^1^H-NMR) spectroscopy in Nanfang Hospital between January 1, 2017, and October 31, 2018, were recruited. The Least Absolute Shrinkage and Selection Operator (LASSO) regression model was used to analyze variables selected by univariate analysis for OS. An index of plasma lipid metabolism profiles, named plasma lipid metabolism index (PLMI), was constructed by variables' coefficients in LASSO regression to explore its association with OS and its role in the prediction model.

**Results:**

A total of 158 GC patients were included in this study. Four of the 110 lipid profiles, including LDL-5 Apo-B, LDL-4 Cholesterol, HDL-4 Apo-A2, and HDL-4 Free Cholesterol, were selected to construct the PLMI. The optimal cut-off value of PLMI for OS was used to classify the population into two subgroups, the high PLMI group (≥ − 0.163) and the low PLMI group (< − 0.163). The high PLMI group had a shorter OS (*p* = 0.0034) and was the independent risk factor for OS (Hazard Ratio = 2.13, 95% Confidence Interval (CI): 1.07–4.22, *p* = 0.031) after adjusting for perineural invasion and tumor stage. In subsets of the I–III stage and treating postoperative chemotherapy, high PLMI also had an unfavorable correlation with OS (*p* = 0.016 and *p* = 0.0086, respectively). The nomogram prediction models of both the training cohort and validation cohort showed good calibration and discrimination with the concordance indexes of 0.806 (95% CI, 0.732–0.880) in the training cohort and 0.794 (95% CI, 0.725–0.862) in the validation cohort.

**Conclusions:**

This study found that the index derived from the LDL-5 Apo-B, LDL-4 Cholesterol, HDL-4 Apo-A2, and HDL-4 Free Cholesterol, was significantly associated with overall survival, suggesting that regulating lipid metabolisms might improve the prognosis for GC patients.

**Supplementary Information:**

The online version contains supplementary material available at 10.1186/s12986-023-00728-1.

## Introduction

Lipid metabolism dysregulation is a significant alteration in the development of cancer, involving proliferation, invasion, metastasis, and therapeutics in cancer due to cancer cells harnessing lipid metabolism to get the elements needed to make biological membranes, energy, as well as signaling molecules [1]. Current researches suggest that lipid metabolism changes in cancer are capable of mediating antitumor drug resistance by up-regulating lipogenesis and lipolytic enzyme expression [2]. In a mouse model, tumor tissues were reported to be able to increase the expression of proteins involved in lipid metabolism in gastric cancer (GC) [3, 4]. Previous studies have mostly focused on the standard lipid risk markers, such as triglycerides, high-density lipoprotein (HDL), low-density lipoprotein (LDL), and very low-density lipoprotein (VLDL) [5–8]. Numerous types of research have been published to date to clarify the roles of plasma lipid profiles in various cancers; however, inconsistent and conflicting results were found, indicating that more research is needed to fully understand the function of lipid metabolism in cancers [9–14]. Part of the contentious findings can be attributed to the widely used clinical blood lipid profile test, which only measures total triglycerides, total cholesterol, LDL, HDL, HDL-cholesterol, and LDL-cholesterol. A thorough understanding of the plasm lipid subfractions may contribute to better deciphering the intricate biochemical mechanisms of lipid metabolism dysregulation that occurs in GC patients.

Proton nuclear magnetic resonance (^1^H-NMR) detection is an advanced technology to analyze plasma lipid metabolism profile, enabling the distinction of different lipid subfractions, which are dependent on different densities, particle sizes, and chemical heterogeneity [15–18]. Currently, the technology of ^1^H-NMR spectroscopy is gradually applied to investigate the development and clinical outcomes in the field of cancer research. Chan et al. carried out a study by measuring various urinary metabolomic components among patients with GC utilizing ^1^H-NMR spectroscopy to exploit a discriminatory urinary metabolomic profile to distinguish GC from healthy patients and benign gastric diseases, which constructed a discriminatory model that had a 0.95 area under the receiver operator characteristic curve (ROC) [19]. Another study measured approximately 1,000 plasma metabolites in patients with GC on an MS metabolomics platform, among which 11 lipid-associated metabolites were associated with GC risk [20]. Although the lipid metabolism profile has shown a significant role in diagnosis and predicting the risk of GC according to previous studies, the relationship between the survival outcomes and plasm lipid metabolism profiles based on ^1^H-NMR spectroscopy remains unexplored.

In the present study, we retrospectively collected the blood samples from GC patients treated in Nanfang Hospital between January 1, 2017, and October 31, 2018, and measured their plasm lipid metabolism profiles using ^1^H-NMR spectroscopy. We sought to investigate the general and particular functions of comprehensive plasm lipid metabolism profiles in predicting the clinical outcomes in GC patients undergoing gastrectomy.

## Methods and materials

### Study population

This study retrospectively collected the key clinical information of GC patients getting plasm ^1^H-NMR spectroscopy detection between January 1, 2017, and October 31, 2018, in Nanfang Hospital, Southern Medical University, Guangzhou, China.

### Collection of clinical characteristic data

The information gathered for this investigation comprised gender, age, clinical characteristics including body mass index (BMI), Lauren type, tumor location, tumor differentiation, lymph node metastasis, depth of invasion, distant metastasis, immunohistochemical features including perineural invasion (S-100), lymphatic invasion (D2-40), venous invasion (CD31), as well as human epidermal growth factor receptor 2 (HER2), serum tumor markers including preoperative carcinoembryonic antigen (CEA) and preoperative cancer antigen 19-9 (CA19-9), serum lipid metabolomics data and follow-up information. The 8^th^ edition of the AJCC Cancer Staging Manual of the American Joint Committee on Cancer was used to identify the pathological stage of gastric cancer [21]. The primary outcome, overall survival (OS), was calculated as the interval between the date of surgery and the date of all-cause death.

### Plasma sample collection

Plasma was obtained from blood samples retrieved from GC patients. Blood samples were collected in 5 mL anticoagulant citrate dextrose tubes and centrifuged at 1700 rpm for 13 min at 4 to 8 °C. About 400 μL of supernatant plasma was collected to cryovials and then saved at − 80 °C waiting for ^1^H-NMR analysis.

### ^1^H-NMR analyses of plasma lipid metabolism profiles

Plasma lipid metabolism profiles were measured by ^1^H-NMR spectroscopy. Briefly, 350 μL of plasma samples after thawing were well mixed with an equal amount of sodium phosphate buffer. by vortexing for about 30 s before being transferred for NMR analysis. ^1^H-NMR spectroscopy was obtained by employing a 310 K and 600.13 MHz proton Larmor frequency Bruker 600 MHz NMR spectrometer.

The Bruker IVDr lipoprotein subclass analysis platform was used to quantify lipids and their subclassifications. The 110 lipid parameters include triglycerides, cholesterol, Apo- B, Apo-A1, Apo-A2, HDL, LDL, VLDL, intermediate-density lipoprotein (IDL), as well as subfractions of each lipoprotein, subdivided according to their density and their concentrations of triglycerides, cholesterol, phospholipids, free cholesterol, Apo-B, Apo-A1, and Apo-A2. HDL was divided into HDL 1-4, LDL into LDL 1-6, and VLDL into VLDL 1-6 for each subfraction with increasing density (Additional file 3: Table S1) [17, 22].

### Statistical analysis

The univariate analysis of Cox regression was first used to estimate the risk of 110 biomarkers of plasm lipid metabolism profiles for OS and the variables with p < 0.05 were included in the analysis of the Least Absolute Shrinkage and Selection Operator (LASSO) regression model with tenfold cross-validation, in which the selected variables underwent multicollinearity test before being used to construct a plasm lipid metabolism index (PLMI). We figured out the optimal cut-off value of PLMI for OS according to the Youden index. Based on the above optimal cut-off value, patients were divided into two groups, high PLMI, and low PLMI groups. The Kaplan–Meier curve was applied to distinguish survival differences between the high PLMI group and the low PLMI group. Multivariate analysis was further used to adjust the confounding effects of clinical characteristics. Furthermore, we explored the association of PLMI with OS in subgroups of tumor stages and postoperative chemotherapy. Finally, we estimated the role of PLMI in improving prediction models for OS. All the analyses were conducted by SPSS version 25.0 (IBM), Graphpad prism 9.0, and R version 4.1.0. *p* < 0.05 was identified as statistical significance.

## Results

### Baseline data

The study flowchart is shown in Additional file 1: Fig. S1. A total of 223 patients with GC underwent ^1^H-NMR measurement. One hundred and fifty-eight GC patients with gastrectomy were selected as the study cohort after excluding 20 patients who had not undergone surgery and 45 patients who underwent exploratory surgery but not gastrectomy.

The mean age of patients was 59 years and 54 (34%) were women and 104 (66%) were men. One hundred and forty-three had undergone radical gastrectomy and 15 had undergone palliative gastrectomy. About half (47%) of them had the tumor at a lower part and about 4% of them had tumors that invaded multiple locations. The rate of death in the cohort was 36.7% (58/158). The primary cause of death was cancer-related death. More details of these clinical features can be seen in Table 1.

**Table 1:** The clinical characteristics of GC patients with gastrectomy

Characteristics	N	%
Total	158	100
Age (years, mean ± standard deviation [SD])	59.06 ± 11.63	
Gender		
Male	104	66
Female	54	34
BMI (kg/m^2^, mean, SD)	22.20 ± 3.22	
Tumor location		
Upper	45	28
Middle	33	21
Low	74	47
Across	6	4
Lauren Type		
Intestinal	54	34
Diffuse	75	16
Mix	26	47
Missing	3	2
Tumor Differentiation		
High	7	4
Moderate	30	19
Poor	119	75
Missing	2	1
Depth of tumor invasion		
pT1	21	13
pT2	28	18
pT3	36	23
pT4	73	46
Lymph node metastasis		
pN0	52	33
pN1	23	15
pN2	24	15
pN3	59	37
Distant metastasis		
M0	143	91
M1	15	9
Postoperative chemotherapy		
Yes	98	62
No	60	38
pTNM stage		
I	36	23
II	36	23
III	71	45
IV	15	9
Perineural invasion (S-100)		
Positive	101	64
Negative	52	33
Missing	5	3
Lymphatic invasion (D2-40)		
Positive	81	51
Negative	72	46
Missing	5	3
Venous invasion (CD31)		
Positive	68	43
Negative	85	54
Missing	5	3
HER2		
0	83	53
1 +	41	26
2 +	22	14
3 +	8	5
Missing	4	3
Preoperative CEA (ug/L)		
≤ 5	130	82
> 5	24	15
Missing	4	3
Preoperative CA199 (U/ml)		
≤ 37	139	88
> 37	15	9
Missing	4	3

### The construction of PLMI

**Fig. 1:**
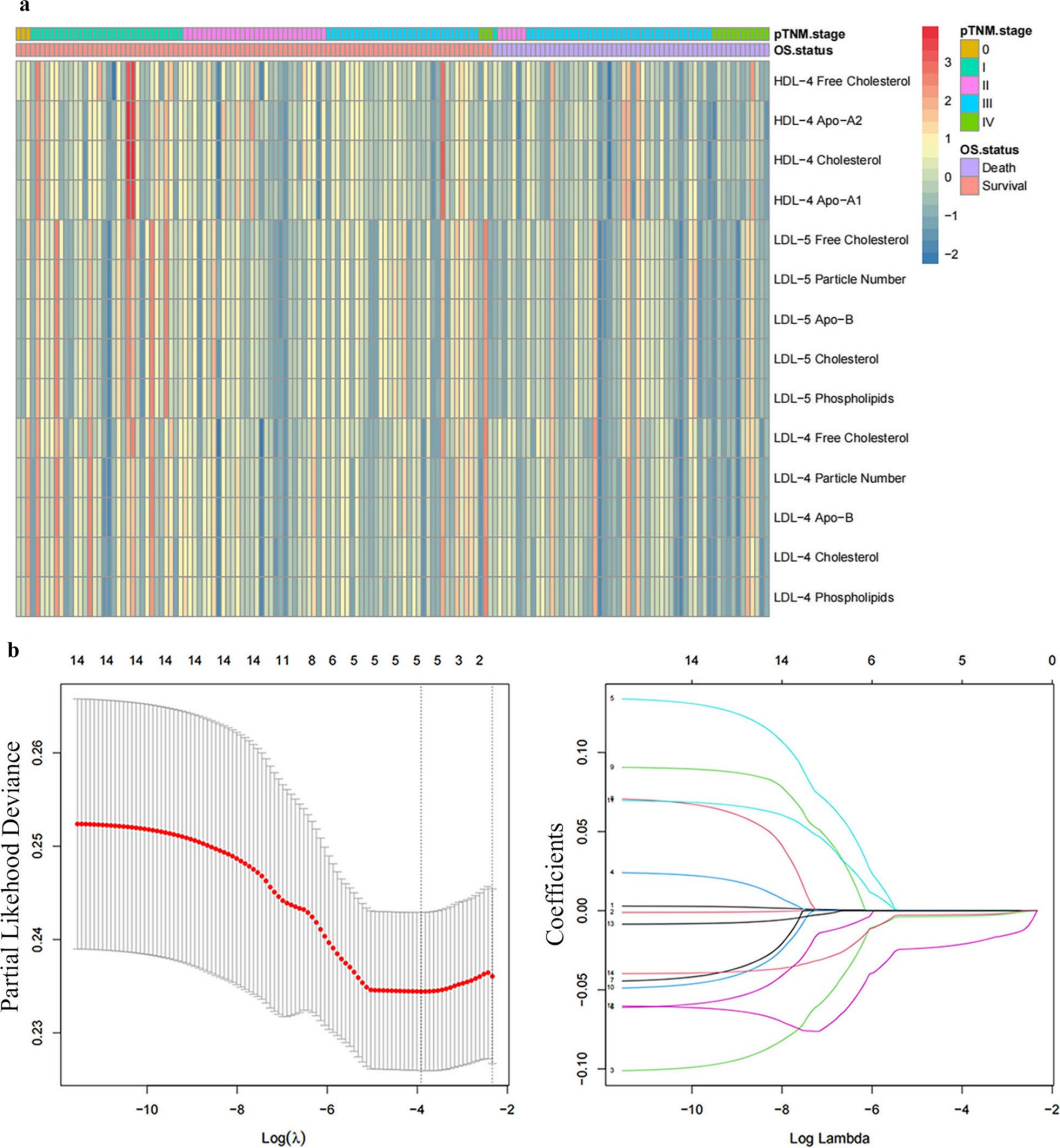
The analysis of 14 variables selected by univariate analysis for OS. **a** Heatmap of levels of 14 lipid metabolism biomarkers normalized to z-score associated with tumor stage and OS status. **b** LASSO Cox regression with tenfold cross-validation and 5 variables were identified out of the 14 variables

The Cox regression was firstly used to analyze the prognostic significance of each plasma lipid metabolism biomarker (110 variables) for OS, among which 14 variables **(**Additional file 3: Table S1) including LDL-5 Particle Number (*p* = 0.04), LDL-4 Particle Number (*p* = 0.03), LDL-5 Cholesterol (*p* = 0.04), LDL-4 Cholesterol (*p* = 0.02), LDL-5 Free Cholesterol (*p* = 0.03), LDL-4 Free Cholesterol (*p* = 0.04), LDL-5 Phospholipids (*p* = 0.04), LDL-4 Phospholipids (*p* = 0.02), LDL-5 Apo-B (*p* = 0.04), HDL-4 Free Cholesterol (*p* = 0.02), LDL-4 Apo-B (*p* = 0.03), HDL-4 Cholesterol (*p* = 0.04), HDL-4 Apo-A1 (*p* = 0.03), and HDL-4 Apo-A2 (*p* = 0.02) showed a significant association. The heatmap of 14 lipid metabolism biomarkers normalized to z-score associated with tumor stage and OS status was shown in Fig. 1 a and they were selected to further analyze within LASSO Cox regression with tenfold cross-validation. Five variables were identified out of the 14 variables (Fig. 1b). Among the above 5 variables, the LDL-5 particle number was excluded after the multicollinearity test for OS (Additional file 3: Table S2). The specific coefficients of the remaining 4 variables selected to construct the PLMI were shown in Additional file 2: Fig. S2. A risk index of PLMI was calculated using a formula derived from the levels of the remaining 4 variables weighted by their regression coefficients. PLMI = (− 0.004 × *LDL-4 Cholesterol*) + (− 0.000004 × *LDL-5 Apo-B*) + (− 0.003 × *HDL-4 Apo-A2*) + (− 0.02 × *HDL-4 Free Cholesterol*).

### Association of PLMI with OS

**Fig. 2:**
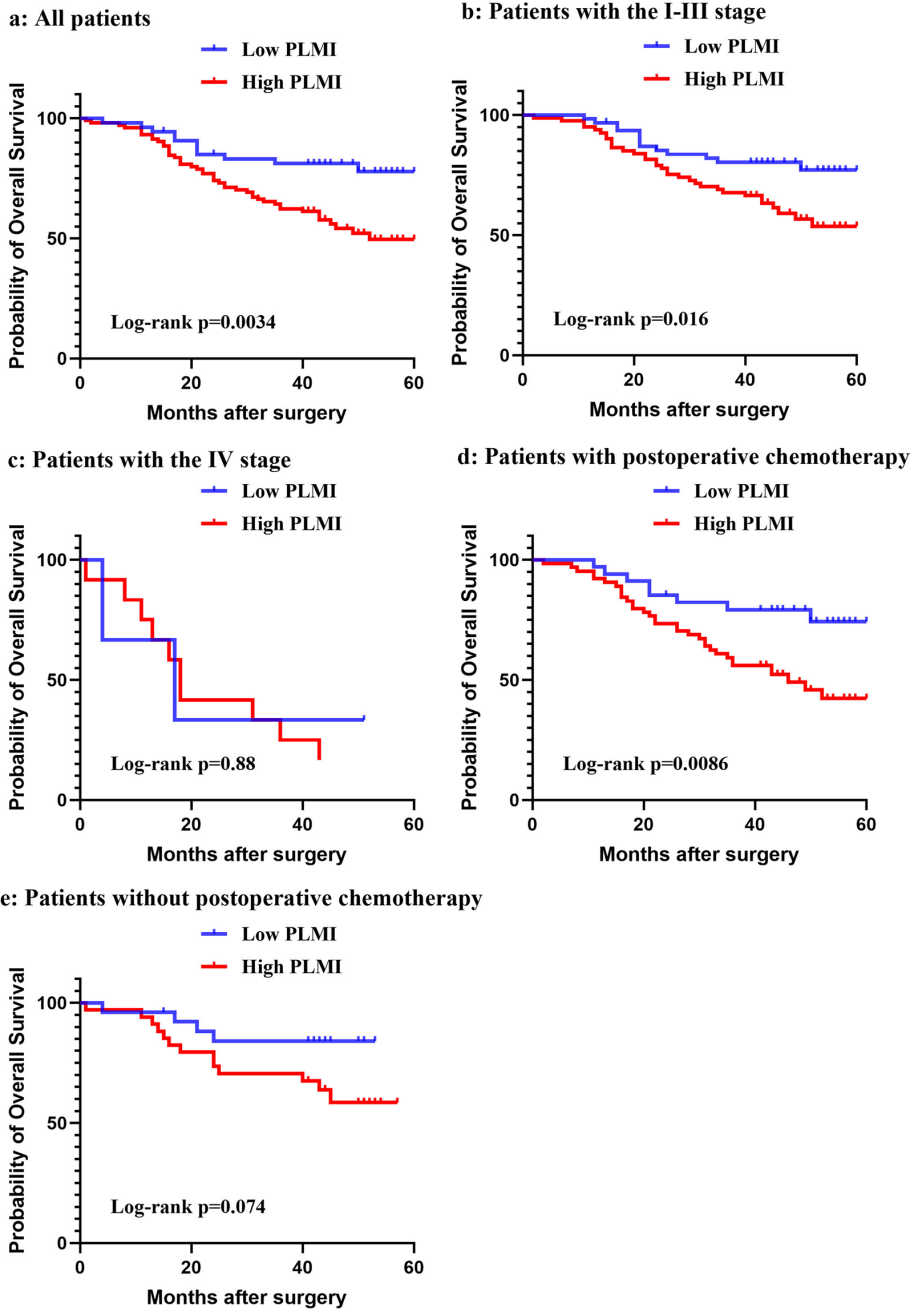
The survival analysis. **a** The OS analysis of the low PLMI and high PLMI in the whole population. **b** The OS analysis of the low PLMI and high PLMI in the subgroup of the I-III stage. **c** The OS analysis of the low PLMI and high PLMI in the subgroup of the IV stage. **d** The OS analysis of the low PLMI and high PLMI in the subgroup of patients with postoperative chemotherapy. **e** The OS analysis of the low PLMI and high PLMI in the subgroup of patients without postoperative chemotherapy

The optimal cut-off value of PLMI for OS was -0.163. The population was classified into two groups relying on the optimal cut-off value, the high PLMI group (≥ − 0.163) and the low PLMI group (< − 0.163). There was a huge difference in OS between the two groups (*p* = 0.0034, Fig. 2a), and in the subgroup of patients with the I–III stage (*p* = 0.016, Fig. 2b) while there was no difference in the subgroup of the IV stage patients (*p* = 0.88, Fig. 2c). In the subgroup of patients with postoperative chemotherapy, the high PLMI group had a shorter OS (*p* = 0.0086, Fig. 2d), but in patients without postoperative chemotherapy, the difference between low PLMI and high PLMI in OS was not significant (*p* = 0.074, Fig. 2e).

To estimate the impact of demographic and clinical characteristics on PLMI, we further carried out univariate and multivariate analyses (Table 2). In the univariate analysis, across tumor location (HR = 3.90, 95% CI: 1.76–8.64, *p* = 0.001), pTNM stage of the IV (HR = 4.50, 95% CI: 2.37–8.57, *p* < 0.001), positive perineural invasion (HR = 9.19, 95% CI: 3.32–25.44, *p* < 0.001), positive lymphatic invasion (HR = 2.69, 95% CI: 1.52–4.76, *p* = 0.001), positive venous invasion (HR = 2.32, 95% CI: 1.36–3.95, *p* = 0.002), preoperative CEA levels > 5 ug/L (HR = 2.88, 95% CI: 1.45–5.73, *p* = 0.003), preoperative CA199 levels > 37 U/ml (HR = 2.14, 95% CI: 1.17–3.91, *p* = 0.01), high PLMI (HR = 2.56, 95% CI: 1.33–4.95, *p* = 0.005) were risk factors for OS. Variables with *p* < 0.05 were included for multivariate analysis, in which high PLMI (HR = 2.13, 95% CI: 1.07–4.22, *p* = 0.03), positive perineural invasion (HR = 6.01, 95% CI: 2.08–17.35, *p* = 0.001), pTNM stage of the IV (HR = 2.70, 95% CI: 1.30–5.63, *p* = 0.008) were identified as risk factors for OS in GC with gastrectomy.

**Table 2:** The univariate and multivariate analyses of OS

Variables	Univariate analysis		Multivariate analysis	
	Hazard Ratio	*p* value	Hazard Ratio	*p* value
Age (years)	1.00 (0.98–1.03)	0.75	–	–
Gender (Male/Female)	1.32 (0.77–2.33)	0.34	–	–
BMI (kg/m^2^)	0.95 (0.87–1.03)	0.23	–	–
Tumor location (Across/others)	3.90 (1.76–8.64)	**0.001**	1.49 (0.58–3.88)	0.41
Lauren Type				
Intestinal	ref			
Diffuse	1.43 (0.79–2.60)	0.24	–	–
Mix	1.51 (0.71–3.23)	0.28	–	–
Tumor Differentiation				
High	ref			
Moderate	-	0.91	–	–
Poor	-	0.91	–	–
Postoperative chemotherapy				
Yes/No	1.49 (0.85–2.63)	0.17	–	–
pTNM stage (IV/ I-III)	4.50 (2.37–8.57)	** < 0.001**	2.70 (1.30–5.63)	**0.008**
Perineural invasion (Positive/Negative)	9.19 (3.32–25.44)	** < 0.001**	6.01 (2.08–17.35)	**0.001**
Lymphatic invasion (Positive/Negative)	2.69 (1.52–4.76)	**0.001**	1.10 (0.49–2.47)	0.82
Venous invasion (Positive/Negative)	2.32 (1.36–3.95)	**0.002**	1.64 (0.78–3.45)	0.20
HER2 (3 + /0–2 +)	1.10 (0.34–3.50)	0.88	–	–
Preoperative CEA (ug/L, > 5/ ≤ 5)	2.88 (1.45–5.73)	**0.003**	1.83 (0.83–4.02)	0.13
Preoperative CA199 (U/ml, > 37/ ≤ 37)	2.14 (1.17–3.91)	**0.01**	1.45 (0.73–2.85)	0.29
Group (High PLMI/Low PLMI)	2.56 (1.33–4.95)	**0.005**	2.13 (1.07–4.22)	**0.03**

The variables with the significance of bold values in univariate analyses have p values < 0.05 and were included in multivariate analyses. The variables with the significance of bold values in multivariate analyses were independent risk factors for OS

### The prediction models for OS

**Fig. 3:**
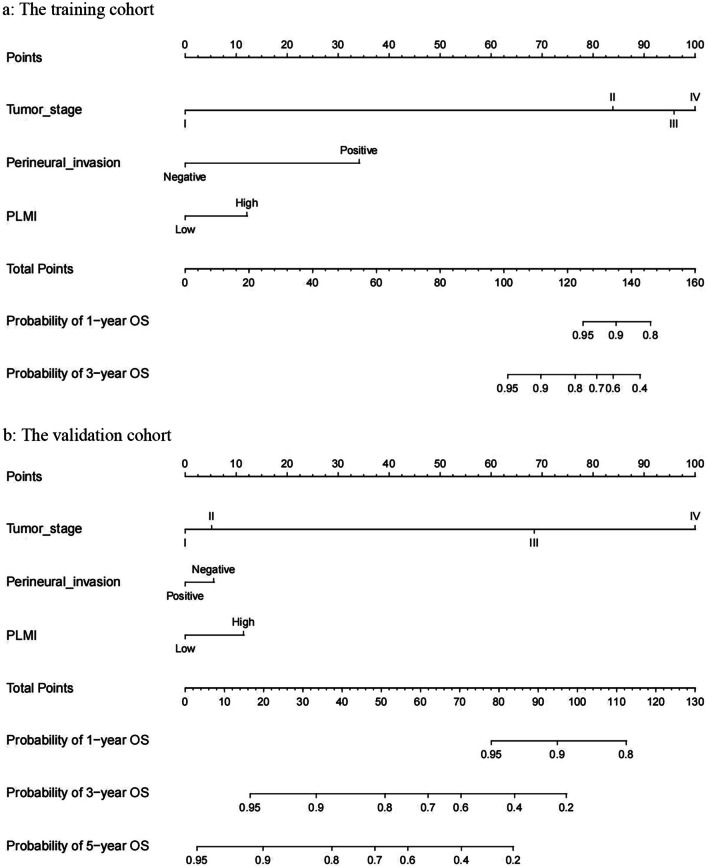
Nomograms for OS prediction. **a** The nomogram for the training cohort in all populations; **b** The nomogram for the validation cohort in all populations

**Fig. 4:**
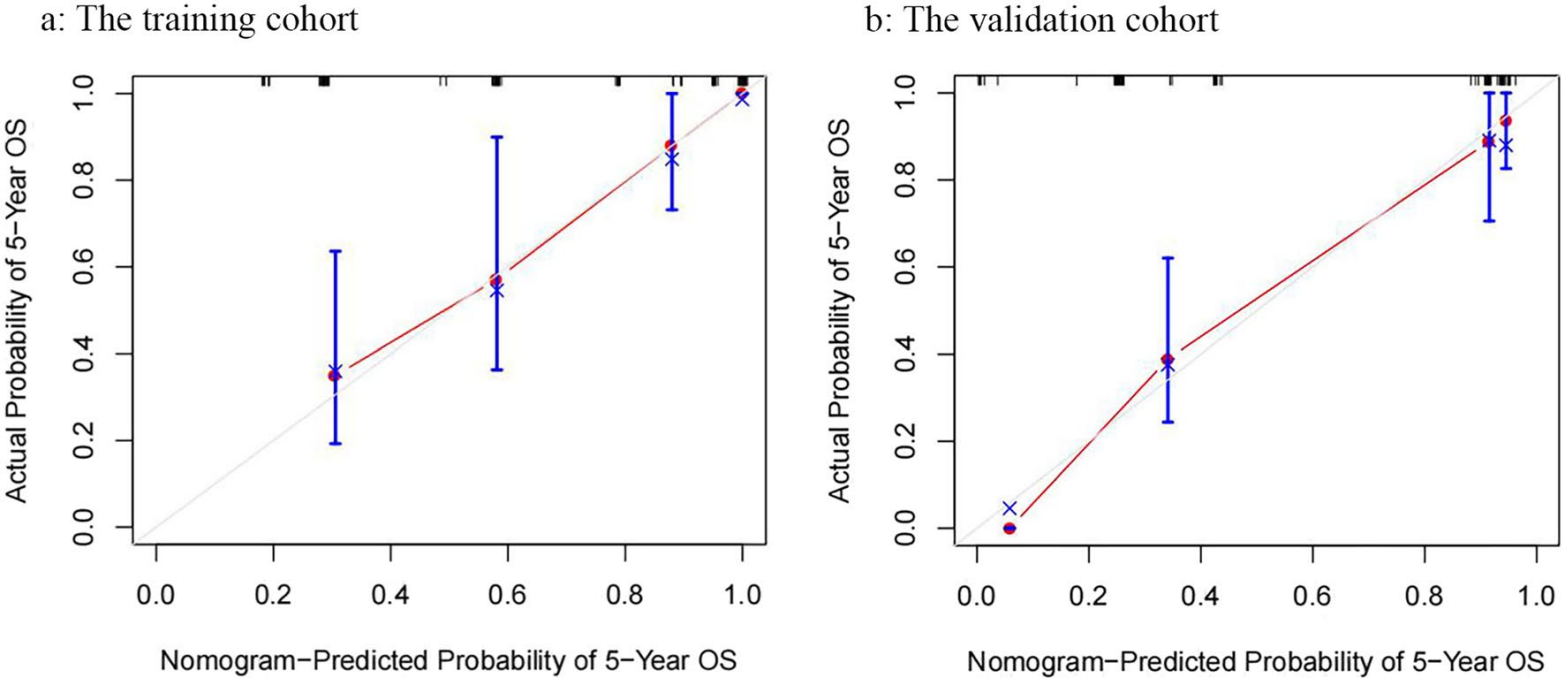
Calibration curves of the Nomograms. **a** The calibration curve for the training cohort; **b** The calibration curve for the validation cohorts. (A perfect calibration model would have a dotted line at the 45-degree line, with predicted probability mirroring actual results.)

The statistically significant variables in multivariate analysis including PLMI, perineural invasion, and tumor stage were applied to create a nomogram to predict the OS. Patients were randomly divided equally into two cohorts, the training cohort, and the validation cohort. There was no significant difference between the two cohorts (Additional file 3: Table S3). The models of the nomogram for the training cohort and the validation cohort were shown in Fig. 3a and b, respectively. Model performance was validated for calibration and discrimination using boot-strapping with 1000 resamples. The calibration curves illustrated good consistency between observed and predicted outcomes in both the training cohort (Fig. 4a) and the validation cohort (Fig. 4b). The model also resulted in a concordance index (C-index) of 0.806 (95% CI, 0.732–0.880) and 0.794 (95% CI, 0.725–0.862) for the training and validation cohorts, respectively.

## Discussion

### The main findings of our study

In this study, a plasma lipid metabolism index, PLMI, was constructed by 4 lipid variables (LDL-5 Apo-B, LDL-4 Cholesterol, HDL-4 Free Cholesterol, HDL-4 Apo-A2), that was capable of predicting survival for GC patients with gastrectomy. Our results showed that high PLMI was associated with shorter OS and was an independent risk factor for OS. Especially patients with high PLMI seemed to benefit less from postoperative chemotherapy with worse OS. Furthermore, we found that the prediction model based on PLMI, tumor stage, and perineural invasion for OS had great power.

### Previous studies about HDL-4 Free Cholesterol, LDL-4 Cholesterol, HDL-4 Apo-A2, and LDL-5 Apo-B

Rare researchers are investigating the role of HDL-4 Free Cholesterol, LDL-4 Cholesterol, HDL-4 Apo-A2, and LDL-5 Apo-B in predicting prognosis for GC. It was reported that higher HDL cholesterol levels were linked both to a lower risk of developing cancer and to a better likelihood of survival in colorectal cancer [23], similarly to which high levels of HDL- Cholesterol indicated a better prognosis in GC [8, 24]. Although the study regarding the associations of HDL Free Cholesterol with GC has not been reported before, this biomarker was the most important factor in the construction of PLMI. Apo-A2, one of the apolipoproteins associated with HDL, was also studied in carcinoma, showing that plasma expressions of Apo-A2-AT/A and Apo-A2-A/A, subfractions of Apo-A2, were related with a greater probability of developing pancreatic cancer, but no correlation with survival was investigated [25]. More studies are needed to further explore the association of HDL Apo-A2 with GC. In addition, increased LDL Cholesterol levels are linked to the risk of gastric cancer but not OS [5]. Apo-B, the only apolipoprotein associated with LDL, is regarded to be linked to the risk of development and unfavorable prognosis in cancer [26, 27]. Although LDL—Cholesterol and LDL Apo-B seemed to be unfavorable biomarkers for cancer, LDL-4 Cholesterol, and LDL-5 Apo-B, as subfractions of LDL Cholesterol and LDL Apo-B, respectively, were favorable factors in our study, which indicates that different densities of LDL Apo-B and LDL Cholesterol may play a different role in prognosis for GC. According to our results, while the general classifications of various lipids did not show robust relationships with survival in GC, the PLMI constructed by the subfractions of lipoproteins showed a significant correlation with prognosis for GC patients, which indicates that subfractions of lipoproteins may have great potential in cancer investigation.

### The relationship between lipid metabolism and antitumor drug resistance in GC

Lipid metabolism has a significant relationship with antitumor drug resistance in GC. Cholesterol affects cancer cell signaling pathways, such as NF-kB [28], oxysterol-binding protein-like protein three [29], R- Ras/Akt [29], HMGCR [30], and Ar inhibitors [31], and so on, thus promoting chemotherapeutic drug resistance in GC. Furthermore, cholesterol can mediate the chemotherapy resistance under platinum-based chemotherapy by regulating ABCG2 expression. Wu et al. revealed that patients who presented with quick chemoresistance had an increased level of plasma cholesterol and their tumor tissue had enhanced ABCG2 expression in lung adenocarcinoma [32]. By targeting Akt/b-catenin and Ras/MEK/ERK signaling pathways, phosphatidylcholine metabolism also contributes to addressing medication resistance [33]. Notably, lipid reprogramming changes intercellular lipid metabolism and immune cells' pharmaceutical resistance in the tumor microenvironment [2]. In present study, we interestingly discovered that GC patients with unfavorable plasma lipid metabolism profiles allowed by the ^1^H-NMR platform had a significantly shorter OS under postoperative chemotherapy, further supporting the hypothesis that lipid metabolism may play an important role in antitumor drug resistance in GC.

### The advantages of ^1^H-NMR technology

^1^H-NMR technology and mass spectrometry (MS) are the two most successful methods of determining the metabolic profiles of an organic liquid [34]. ^1^H NMR spectroscopy has several advantages over MS for metabolic applications, including easy preparation, non-destructive analysis, in*-vivo* application*,* the ability to detect a variety of substances, and analysis of the structure of unknown compounds [35]. ^1^H-NMR is effective at tracing metabolic activities and fluxes with the usage of isotope labeling. ^1^H-NMR data provide a higher degree of repeatability over a larger dynamic range and have a track record of converting in-vitro results into *Vivo* clinical applications [34]. In the present investigation, we utilized the ^1^H-NMR spectroscopy to test blood samples extracted from GC patients and explored the association of the lipid metabolism profiles with OS. Although the study about plasma lipid metabolism profiles based on ^1^H-NMR technology in GC has not been found yet, the application of ^1^H-NMR technology focusing on other metabolomic aspects in GC was reported previously. In a study, patients’ urine was collected to be measured by ^1^H-NMR to identify whether GC has a unique urinary metabolomic profile compared with benign gastric disease and healthy patients, showing that a score based on 2-hydroxyisobutyrate, 3-indoxylsulfate, and alanine selected by LASSO regularised logistic regression produced a good discriminatory model with an area under the ROC of 0.95 [19]. The other study exerted the ^1^H-NMR technology to detect metabolic profiling of tissues from GC patients and healthy patients, finding that PLS-DA (partial least-squares discriminant analysis) models based on 48 endogenous distinguishing metabolites including glycolysis, glutaminolysis, amino acids, and choline showed adequate discrimination between cancer tissues and normal controls (36). Accordingly, previous studies involving ^1^H-NMR technology in GC research mainly focused on diagnostic value for GC by detecting urine or tissue rather than blood, whereas the present study especially explored the relationship between the plasma lipid metabolism profiles and GC prognosis, helping to further investigate the metabolomic potential in GC.

### Highlights

Our study has some highlights. Firstly, we applied an advanced technology of ^1^H-NMR spectroscopy in our study, with the help of which we were capable of testing 110 parameters of lipids and their specific subfractions, enabling us to conduct more comprehensive research concerning plasma lipid metabolism profiles for GC patients with gastrectomy. Secondly, we construct a good index of PLMI that was capable of demonstrating the characteristics of plasm lipid metabolism profiles. Thirdly, while the previous research based on ^1^H-NMR mainly focused on the risk and diagnosis value for cancers, our study was exceptionally designed to explore the role of lipid metabolism profiles in prognosis for GC by including a total of 158 GC patients with gastrectomy with 5-year follow-up, which could be able to strongly support our conclusion.

### Limitations

There are also some limitations in our study. Firstly, due to the study's retrospective methodology, it was prone to some inherent biases, thus we would like to conduct a prospective, multicenter study to confirm our conclusion further. Secondly, although the models including PLMI in our study had been validated in the internal validation cohort, it would be better if an external validation cohort were available, which is also the key we attempt to improve in our future research.

## Conclusion

This study found that the index derived from the LDL-5 Apo-B, LDL-4 Cholesterol, HDL-4 Apo-A2, and HDL-4 Free Cholesterol, was significantly associated with overall survival, suggesting that regulating lipid metabolisms might improve the prognosis for GC patients.

## Supplementary Information

**Additional file 1. Fig. S1.** The flowchart of the study.

**Additional file 2. Fig. S2.** The specific coefficients of 4 variables being used toconstruct the PLMI.

**Additional file 3. Supplemental tables. Table S1.** The univariate analysis of lipid metabolism biomarkers for OS; **Table S2.** Multicollinearity test of 5 variables for OS; **Table S3.** The baseline data of the training cohort and the validation cohort.

## Data Availability

Data are available by contacting the corresponding author with reasonable requests.

## Abbreviations

^1^H-NMR: Proton nuclear magnetic resonance
OS: Overall survival
GC: Gastric cancer
LASSO: Least Absolute Shrinkage and Selection Operator
PLMI: Plasma lipid metabolism index
VLDL: Very low-density lipoprotein
LDL: Low-density lipoprotein
HDL: High-density lipoprotein
ROC: Receiver operator characteristic curve
BMI: Body mass index
HER2: Human epidermal growth factor receptor 2
CEA: Carcinoembryonic antigen
CA19-9: Preoperative cancer antigen 19-9
IDL: Intermediate-density lipoprotein
MS: Mass spectrometry
