# Supplementary figures and images for: Association of plasma lipid metabolism profiles with overall survival for patients with gastric cancer undergoing gastrectomy based on 1H-NMR spectroscopy
Source: Nutr Metab (Lond). 2023 Feb 7;20:7. doi: 10.1186/s12986-023-00728-1 (PMC9903497; doi:10.1186/s12986-023-00728-1)

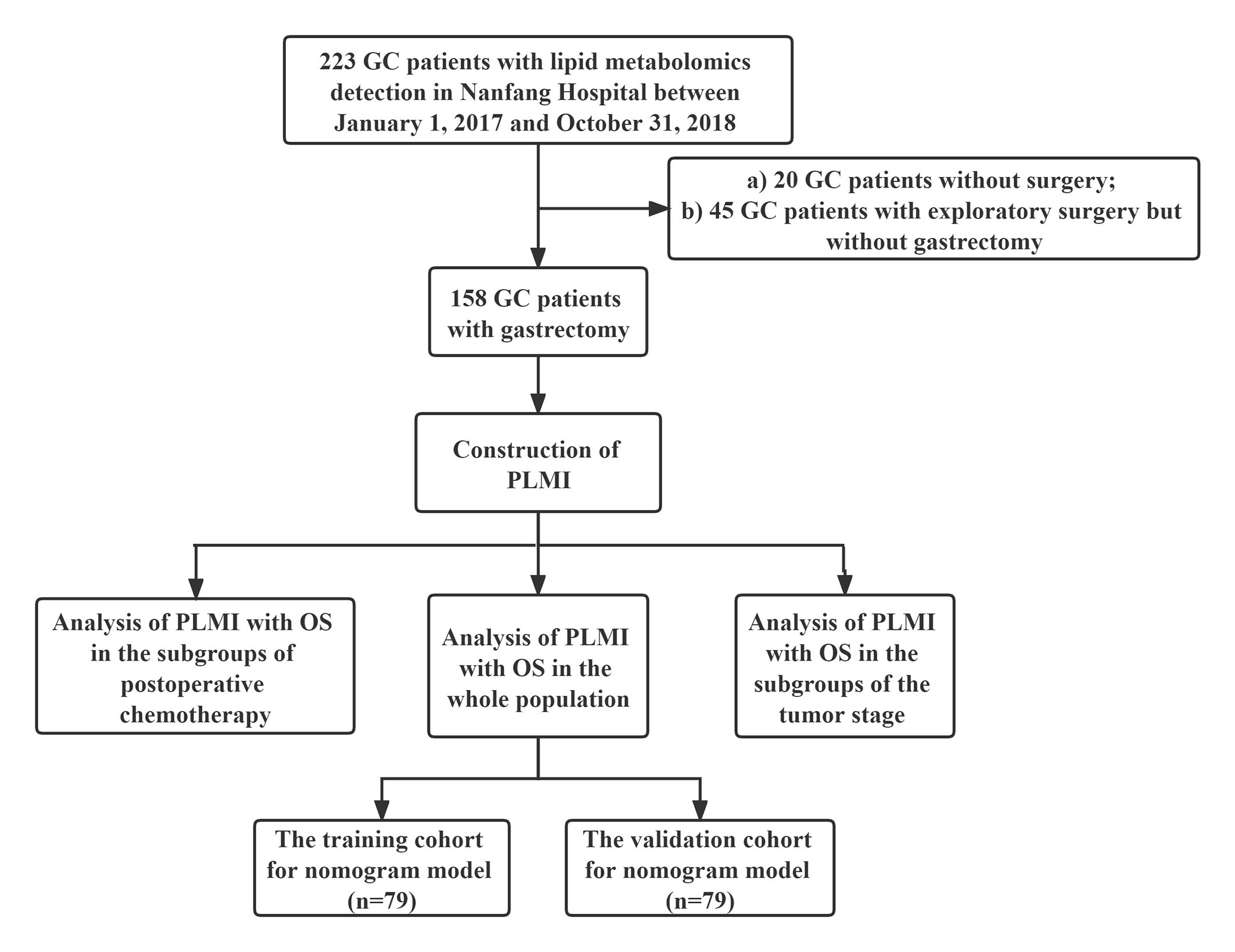

Supplement: Supplementary file 1 — Additional file 1. Fig. S1. The flowchart of the study. [file 12986_2023_728_MOESM1_ESM.jpg]

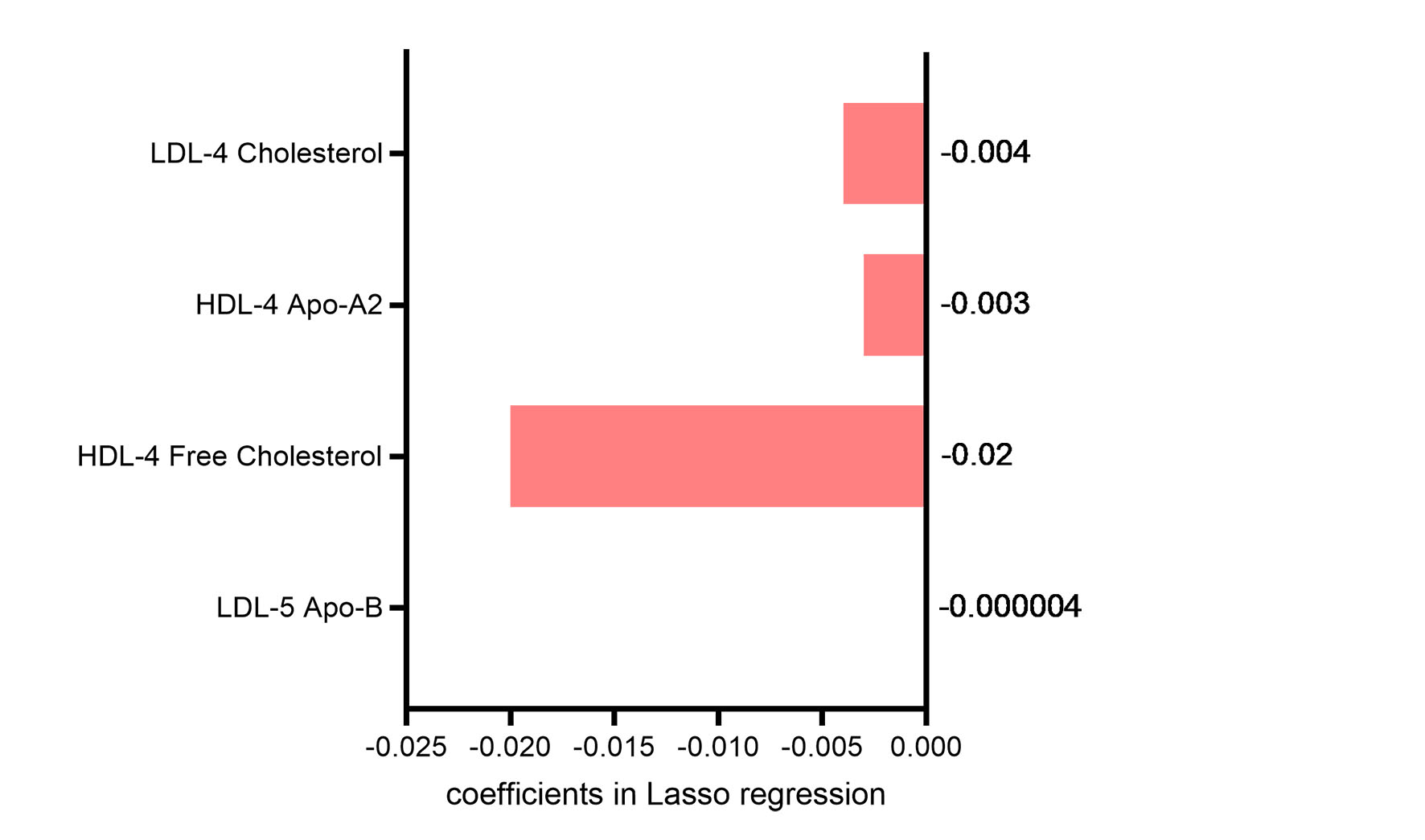

Supplement: Supplementary file 2 — Additional file 2. Fig. S2. The specific coefficients of 4 variables being used toconstruct the PLMI. [file 12986_2023_728_MOESM2_ESM.jpg]
