# Supplementary material for: Association of plasma lipid metabolism profiles with overall survival for patients with gastric cancer undergoing gastrectomy based on 1H-NMR spectroscopy
Source: Nutr Metab (Lond). 2023 Feb 7;20:7. doi: 10.1186/s12986-023-00728-1 (PMC9903497; doi:10.1186/s12986-023-00728-1)
Supplement: Supplementary file 3 — Additional file 3. Supplemental tables. Table S1. The univariate analysis of lipid metabolism biomarkers for OS; Table S2. Multicollinearity test of 5 variables for OS; Table S3. The baseline data of the training cohort and the validation cohort. [file 12986_2023_728_MOESM3_ESM.docx]

**Supplemental Table 1** The univariate analysis of lipid metabolism biomarkers for OS.

| Variables | HR, 95%CI | p-value |
| --- | --- | --- |
| Cholesterols | 1.00(0.99-1.00) | 0.15 |
| Triglycerides | 1.00(1.00-1.00) | 0.93 |
| LDL- Cholesterol | 1.00(0.99-1.00) | 0.08 |
| HDL- Cholesterol | 1.00(0.99-1.01) | 0.56 |
| Apo-A1 | 1.00(0.99-1.00) | 0.45 |
| Apo-A2 | 0.99(0.96-1.00) | 0.11 |
| Apo-B | 0.99(0.98-1.00) | 0.11 |
| Total Particle Number | 1.00(1.00-1.00) | 0.11 |
| VLDL Particle Number | 1.00(1.00-1.00) | 0.74 |
| IDL Particle Number | 1.00(0.99-1.00) | 0.16 |
| LDL Particle Number | 1.00(1.00-1.00) | 0.13 |
| IDL Triglycerides | 1.00(0.97-1.03) | 0.85 |
| LDL Triglycerides | 0.99(0.96-1.03) | 0.71 |
| HDL Triglycerides | 1.02(0.97-1.08) | 0.48 |
| VLDL Triglycerides | 1.00(0.99-1.00) | 0.54 |
| VLDL Cholesterol | 0.99(0.97-1.01) | 0.51 |
| IDL Cholesterol | 0.98(0.95-1.01) | 0.11 |
| VLDL Free Cholesterol | 0.99(0.94-1.04) | 0.64 |
| IDL Free Cholesterol | 0.93(0.86-1.02) | 0.13 |
| LDL Free Cholesterol | 0.98(0.96-1.00) | 0.11 |
| HDL Free Cholesterol | 1.01(0.95-1.07) | 0.79 |
| VLDL Phospholipids | 0.99(0.96-1.02) | 0.45 |
| IDL Phospholipids | 0.97(0.93-1.01) | 0.18 |
| LDL Phospholipids | 0.99(0.98-1.00) | 0.09 |
| HDL Phospholipids | 1.00(0.99-1.01) | 0.90 |
| HDL Apo-A1 | 1.00(0.99-1.00) | 0.42 |
| HDL Apo-A2 | 0.98(0.97-1.00) | 0.12 |
| VLDL Apo-B | 0.99(0.93-1.05) | 0.74 |
| IDL Apo-B | 0.95(0.88-1.02) | 0.16 |
| LDL Apo-B | 0.99(0.98-1.00) | 0.13 |
| LDL-1 Particle Number | 1.00(1.00-1.00) | 0.62 |
| LDL-2 Particle Number | 1.00(1.00-1.00) | 0.47 |
| LDL-3 Particle Number | 1.00(1.00-1.00) | 0.25 |
| **LDL-4 Particle Number** | 1.00(0.99-1.00) | **0.03** |
| **LDL-5 Particle Number** | 1.00(1.00-1.00) | **0.04** |
| LDL-6 Particle Number | 1.00(1.00-1.00) | 0.16 |
| VLDL-1 Triglycerides | 1.00(0.99-1.01) | 1.00 |
| VLDL-2 Triglycerides | 0.99(0.96-1.02) | 0.39 |
| VLDL-3 Triglycerides | 0.98(0.95-1.01) | 0.20 |
| VLDL-4 Triglycerides | 0.97(0.93-1.02) | 0.25 |
| VLDL-5 Triglycerides | 1.18(0.95-1.46) | 0.14 |
| VLDL-1 Cholesterol | 1.01(0.96-1.06) | 0.78 |
| VLDL-2 Cholesterol | 0.96(0.86-1.07) | 0.43 |
| VLDL-3 Cholesterol | 0.94(0.87-1.02) | 0.14 |
| VLDL-4 Cholesterol | 0.95(0.89-1.03) | 0.21 |
| VLDL-5 Cholesterol | 1.20(0.92-1.57) | 0.17 |
| VLDL-1 Free Cholesterol | 0.99(0.88-1.12) | 0.85 |
| VLDL-2 Free Cholesterol | 0.94(0.75-1.17) | 0.56 |
| VLDL-3 Free Cholesterol | 0.91(0.76-1.09) | 0.31 |
| VLDL-4 Free Cholesterol | 0.93(0.80-1.07) | 0.29 |
| VLDL-5 Free Cholesterol | 1.29(0.75-2.21) | 0.36 |
| VLDL-1 Phospholipids | 0.99(0.93-1.05) | 0.80 |
| VLDL-2 Phospholipids | 0.94(0.84-1.05) | 0.27 |
| VLDL-3 Phospholipids | 0.94(0.87-1.03) | 0.17 |
| VLDL-4 Phospholipids | 0.94(0.86-1.04) | 0.24 |
| VLDL-5 Phospholipids | 1.14(0.89-1.47) | 0.30 |
| LDL-1 Triglycerides | 1.09(0.98-1.21) | 0.11 |
| LDL-2 Triglycerides | 1.06(0.81-1.39) | 0.65 |
| LDL-3 Triglycerides | 1.21(0.89-1.65) | 0.22 |
| LDL-4 Triglycerides | 0.91(0.72-1.16) | 0.44 |
| LDL-5 Triglycerides | 0.91(0.77-1.08) | 0.30 |
| LDL-6 Triglycerides | 0.91(0.79-1.05) | 0.19 |
| LDL-1 Cholesterol | 1.00(0.97-1.03) | 0.99 |
| LDL-2 Cholesterol | 0.99(0.96-1.02) | 0.39 |
| LDL-3 Cholesterol | 0.98(0.95-1.01) | 0.20 |
| **LDL-4 Cholesterol** | 0.96(0.94-0.99) | **0.02** |
| **LDL-5 Cholesterol** | 0.98(0.96-1.00) | **0.04** |
| LDL-6 Cholesterol | 0.99(0.97-1.00) | 0.12 |
| LDL-1 Free Cholesterol | 0.98(0.90-1.08) | 0.75 |
| LDL-2 Free Cholesterol | 0.95(0.88-1.04) | 0.27 |
| LDL-3 Free Cholesterol | 0.95(0.86-1.05) | 0.32 |
| **LDL-4 Free Cholesterol** | 0.90(0.81-0.99) | **0.04** |
| **LDL-5 Free Cholesterol** | 0.91(0.84-0.99) | **0.03** |
| LDL-6 Free Cholesterol | 0.95(0.89-1.01) | 0.12 |
| LDL-1 Phospholipids | 1.01(0.95-1.08) | 0.80 |
| LDL-2 Phospholipids | 0.98(0.92-1.04) | 0.44 |
| LDL-3 Phospholipids | 0.96(0.90-1.02) | 0.21 |
| **LDL-4 Phospholipids** | 0.93(0.88-0.99) | **0.02** |
| **LDL-5 Phospholipids** | 0.95(0.91-1.00) | **0.04** |
| LDL-6 Phospholipids | 0.97(0.94-1.01) | 0.14 |
| LDL-1 Apo-B | 1.02(0.95-1.09) | 0.62 |
| LDL-2 Apo-B | 0.98(0.92-1.04) | 0.47 |
| LDL-3 Apo-B | 0.97(0.91-1.03) | 0.25 |
| **LDL-4 Apo-B** | 0.94(0.90-0.99) | **0.03** |
| **LDL-5 Apo-B** | 0.97(0.94-1.00) | **0.04** |
| LDL-6 Apo-B | 0.98(0.96-1.01) | 0.16 |
| HDL-1 Triglycerides | 1.05(0.96-1.15) | 0.26 |
| HDL-2 Triglycerides | 1.05(0.82-1.36) | 0.68 |
| HDL-3 Triglycerides | 0.99(0.73-1.32) | 0.95 |
| HDL-4 Triglycerides | 0.92(0.74-1.14) | 0.43 |
| HDL-1 Cholesterol | 1.01(0.99-1.03) | 0.56 |
| HDL-2 Cholesterol | 0.99(0.94-1.05) | 0.80 |
| HDL-3 Cholesterol | 0.97(0.91-1.03) | 0.33 |
| **HDL-4 Cholesterol** | 0.96(0.92-1) | **0.04** |
| HDL-1 Free Cholesterol | 1.00(0.92-1.08) | 0.92 |
| HDL-2 Free Cholesterol | 0.92(0.76-1.11) | 0.39 |
| HDL-3 Free Cholesterol | 0.85(0.69-1.05) | 0.12 |
| **HDL-4 Free Cholesterol** | 0.82(0.69-0.97) | **0.02** |
| HDL-1 Phospholipids | 1.01(0.99-1.02) | 0.40 |
| HDL-2 Phospholipids | 1.00(0.96-1.04) | 0.90 |
| HDL-3 Phospholipids | 0.98(0.94-1.03) | 0.46 |
| HDL-4 Phospholipids | 0.96(0.93-1.00) | 0.06 |
| HDL-1 Apo-A1 | 1.00(0.99-1.02) | 0.50 |
| HDL-2 Apo-A1 | 1.00(0.97-1.04) | 1.00 |
| HDL-3 Apo-A1 | 0.99(0.96-1.02) | 0.46 |
| **HDL-4 Apo-A1** | 0.99(0.97-1.00) | **0.03** |
| HDL-1 Apo-A2 | 1.02(0.92-1.13) | 0.75 |
| HDL-2 Apo-A2 | 0.96(0.86-1.07) | 0.44 |
| HDL-3 Apo-A2 | 0.95(0.87-1.03) | 0.2 |
| **HDL-4** **Apo-A2** | 0.96(0.92-0.99) | **0.02** |

**Supplemental Table 2** Multicollinearity test of 5 variables for OS.

| Variables | β | t | p | TOL | VIF |
| --- | --- | --- | --- | --- | --- |
| **LDL-5 Particle Number*^a^*** | -372.556 | -1.578 | 0.117 | 0.0000001106 | 9042422.689 |
| LDL-4 Cholesterol | -0.079 | -0.536 | 0.592 | 0.284 | 3.519 |
| LDL-5 Apo-B | -0.031 | -0.206 | 0.837 | 0.267 | 3.742 |
| HDL-4 Free Cholesterol | -0.087 | -0.427 | 0.67 | 0.149 | 6.706 |
| HDL-4 Apo-A2 | -0.046 | -0.217 | 0.828 | 0.14 | 7.162 |

1. The biomarker of LDL-5 particle number was excluded with TOL <0.1 and VIF >10.

**Supplemental Table 3** The baseline data of the training cohort and the validation cohort.

| Variables | The training cohort | The validation cohort | p-value |
| --- | --- | --- | --- |
| Age (years, mean ± standard deviation [SD]) | 59 ± 12 | 59 ± 11 | 0.76 |
| Gender |  |  | 0.18 |
| Male | 48 | 56 |  |
| Female | 31 | 23 |  |
| BMI (kg/m^2^, mean, SD) | 22.3 ± 3.6 | 22.1 ± 2.8 | 0.71 |
| Tumor location |  |  | 0.17 |
| Upper | 21 | 29 |  |
| Middle | 25 | 14 |  |
| Low | 38 | 41 |  |
| Across | 3 | 5 |  |
| Lauren type |  |  | 0.3 |
| Intestinal | 27 | 27 |  |
| Diffuse | 42 | 33 |  |
| Mix | 10 | 16 |  |
| Missing | 0 | 3 |  |
| Tumor differentiation |  |  | 0.88 |
| High | 3 | 4 |  |
| Moderate | 16 | 14 |  |
| Poor | 60 | 59 |  |
| Missing | 0 | 2 |  |
| pTNM stage |  |  | 0.06 |
| I | 16 | 20 |  |
| II | 21 | 15 |  |
| III | 32 | 39 |  |
| IV | 10 | 5 |  |
| Perineural invasion (S-100) |  |  | 0.65 |
| Positive | 51 | 50 |  |
| Negative | 28 | 24 |  |
| Missing | 0 | 5 |  |
| Lymphatic invasion (D2-40) |  |  | 0.12 |
| Positive | 37 | 44 |  |
| Negative | 42 | 30 |  |
| Missing | 0 | 5 |  |
| Venous invasion (CD31) |  |  | 0.72 |
| Positive | 34 | 34 |  |
| Negative | 45 | 40 |  |
| Missing | 0 | 5 |  |
| HER2 |  |  | 0.74 |
| 0 | 45 | 38 |  |
| 1+ | 21 | 20 |  |
| 2+ | 9 | 13 |  |
| 3+ | 4 | 4 |  |
| Missing | 0 | 4 |  |
| Preoperative CEA (ug/L) |  |  | 0.06 |
| ≤5 | 73 | 66 |  |
| >5 | 4 | 11 |  |
| Missing | 2 | 2 |  |
| Preoperative CA199 (U/ml) |  |  | 0.18 |
| ≤37 | 68 | 62 |  |
| >37 | 9 | 15 |  |
| Missing | 2 | 2 |  |
